# Long-term effects of lumacaftor/ivacaftor on lung abnormalities detected by magnetic resonance imaging in children with cystic fibrosis

**DOI:** 10.1186/s40348-026-00268-7

**Published:** 2026-11-10

**Authors:** Lena Wucherpfennig, Monika Eichinger, Mirjam Stahl, Simon Y. Graeber, Shengkai Zhao, Jaehi Chung, Susanne Hämmerling, Jens-Peter Schenk, Abdulsattar Alrajab, Rory E. Morty, Julian Berges, Hans-Ulrich Kauczor, Marcus A. Mall, Mark O. Wielpütz, Eva Steinke, Olaf Sommerburg

**Affiliations:** 1 Department of Diagnostic and Interventional Radiology, University Hospital Heidelberg, Im Neuenheimer Feld 420, Heidelberg, 69120 Germany; 2 Translational Lung Research Center Heidelberg (TLRC), German Center for Lung Research (DZL), Im Neuenheimer Feld 156, Heidelberg, 69120 Germany; 3 Department of Diagnostic and Interventional Radiology with Nuclear Medicine, Thoraxklinik, University Hospital Heidelberg, Röntgenstr. 1, Heidelberg, 69126 Germany; 4 Department of Pediatric Respiratory Medicine, Immunology and Intensive Care Medicine, Charité-Universitätsmedizin Berlin, Augustenburger Platz 1, Berlin, 13353 Germany; 5 German Center for Lung Research (DZL) associated partner site, Augustenburger Platz 1, Berlin, 13353 Germany; 6 German Center for Child and Adolescent Health (DZKJ) partner site, Augustenburger Platz 1, Berlin, 13353 Germany; 7 Berlin Institute of Health (BIH) at Charité – Universitätsmedizin Berlin, Anna-Louisa-Karsch-Str. 2, Berlin, 10178 Germany; 8 Division of Pediatric Pulmonology & Allergy and Cystic Fibrosis Center, Department of Pediatrics III, University Hospital Heidelberg, Im Neuenheimer Feld 430, Heidelberg, 69120 Germany; 9 Division of Pediatric Radiology, Department of Diagnostic and Interventional Radiology, University Hospital Heidelberg, Im Neuenheimer Feld 420, Heidelberg, 69120 Germany; 10 Department of Translational Pneumology, Heidelberg University Hospital, Im Neuenheimer Feld 156, Heidelberg, 69120 Germany; 11 Cluster of Excellence ImmunoPreCept, Charité - Universitätsmedizin Berlin, Augustenburger Platz 1, Berlin, 13353 Germany; 12 Department of Diagnostic Radiology and Interventional Radiology, University Medicine Greifswald, Ferdinand-Sauerbruch-Strasse 1, Greifswald, 17475 Germany

**Keywords:** Cystic fibrosis, Magnetic resonance imaging, Airway disease, CFTR modulator, Mucus obstruction

## Abstract

**Background:**

In a previous trial, magnetic resonance imaging (MRI) showed stabilisation of structural and functional lung abnormalities in children with cystic fibrosis (CF) after up to 96 weeks of Lumacaftor/Ivacaftor (LUM/IVA) therapy. However, real-world evidence on long-term effects in preschool and school-age children is limited.

**Methods:**

Forty-nine children with CF homozygous for *F508del* (mean age 5.0 ± 3.5years, range 1-12years) underwent chest MRI before (MRI1) and annually up to four years on LUM/IVA (MRI2-5, mean 3.5 ± 1.1 examinations; mean follow-up 2.6 ± 1.1years). Treatment effects were evaluated using the chest MRI score, lung clearance index (LCI) and percent predicted forced expiratory volume in one second (ppFEV1).

**Results:**

MRI global and morphology scores significantly decreased from MRI1 to MRI2 (15.9 ± 6.8 vs. 13.7 ± 6.5, *p* < 0.05; and 11.6 ± 4.8 vs. 10.6 ± 4.8, *p* < 0.05, respectively), while MRI perfusion score showed a non-significant decrease (4.7 ± 2.7 vs. 3.7 ± 2.6, *p* = 0.200). From MRI2 through MRI5, MRI global and perfusion scores remained stable (*p* = 0.308–0.999), whereas MRI morphology score increased at MRI4 and MRI5 to the level of MRI1 (*p* = 0.990 and *p* = 0.999 vs. MRI1). LCI was stable between MRI1 and MRI2 (9.0 ± 2.4 vs. 8.2 ± 2.2, *p* = 0.253) and was lower from MRI3 onward compared to MRI1 (*p* < 0.05), while ppFEV1 was stable (*p* = 0.355–0.999). Improvements in MRI global score between MRI1 and MRI2 correlated with improvements in LCI (*r* = 0.42, *p* < 0.05).

**Conclusions:**

Longitudinal chest MRI demonstrates initial improvement and second-year stabilization of morphological and functional lung abnormalities in preschool and school-age children with CF homozygous for *F508del* under LUM/IVA therapy, with increasing morphological abnormalities toward pre-treatment levels during the third year of therapy.

**Supplementary Information:**

The online version contains supplementary material available at https://doi.org/10.1186/s40348-026-00268-7.

## Introduction

Cystic fibrosis (CF) is an autosomal-recessive disorder caused by mutations in the cystic fibrosis transmembrane conductance regulator (*CFTR*) gene, leading to chronic inflammation with progressive lung destruction [1–3]. The approval of CFTR modulator combination therapy has markedly improved CF treatment compared to symptomatic therapy by improving the processing and transport of CFTR proteins to the cell surface and CFTR channel gating [1, 4]. The dual combination therapy with lumacaftor/ivacaftor (LUM/IVA) was approved for the treatment of children with CF homozygous for the *F508del* mutation aged six years and older in 2016 in the United States and 2018 in the European Union, and for children homozygous for the *F508del* mutation aged between two and six years in 2018 in the United States and 2019 in the European Union. Since approximately 40% of people with CF are homozygous for *F508del* (F/F genotype), therapy with LUM/IVA was a viable option for more than one third of people with CF prior to the approval of triple combination therapy with elexacaftor/tezacaftor/ivacaftor (ETI) [5]. Previous studies demonstrated improvements in clinical parameters such as sweat chloride concentration (SCC), growth parameters and exocrine pancreatic function in children starting LUM/IVA between two and five years of age after 24 to 96 weeks of therapy [6–9]. Moreover, improvements in lung clearance index (LCI) were reported in children aged two years and older within 96 weeks after initiating of LUM/IVA treatment [8–10]. In the last decade, chest magnetic resonance imaging (MRI) in combination with a dedicated MRI scoring system was introduced as a novel, radiation-free non-invasive imaging endpoint studying the progression of structural and functional lung abnormalities and the response to various therapies in patients with CF across all age-groups. MRI was further shown to correlate with lung function parameters such as percent predicted forced expiratory volume in 1 s (ppFEV1) and LCI [10–21]. A previous study in children and young adults with CF using MRI demonstrated improvements in structural and functional lung abnormalities within the first eight to 16 weeks of therapy with LUM/IVA [10]. Furthermore, in a randomized controlled phase 2 trial, positive treatment effects of LUM/IVA on chest MRI score were observed after 48 weeks of therapy in children who started LUM/IVA at the age of two to five years [22]. Moreover, an open-label extension period of further 48 weeks of LUM/IVA showed additional effects on lung abnormalities at MRI with stabilization in the previous verum group and improvements in the MRI score in the previous placebo group [9].

However, long-term treatment effects on lung abnormalities detected by MRI in children treated with LUM/IVA in a real-world setting, particularly beyond two years of therapy, were not reported so far. Moreover, the progression of lung disease at MRI was recently reported for preschool and school-age children with CF without modulator treatment [21]. Therefore, the aim of this longitudinal observational study was to assess the effects of long-term therapy with LUM/IVA on the progression of structural and functional lung abnormalities by annual chest MRI in conjunction with the chest MRI scoring system in 49 preschool and school-age children with CF during longitudinal follow-up, with a mean follow-up duration of 2.6 ± 1.1 years and a maximum follow-up of four years, amounting to a total of 172 MRI examinations.

## Methods

### Study population

This study was approved by the institutional ethics committees (S-211/2011, EA2/016/18) and written informed consent was obtained from all parents or legal guardians. The diagnosis of CF was based on newborn screening (*n* = 22) or clinical symptoms (*n* = 27), and confirmed according to recent guidelines [23, 24]. Forty-nine children with CF homozygous for *F508del* (mean age at baseline MRI 5.0 ± 3.5 years, range 1–12 years), who first started modulator therapy with LUM/IVA between February 2018 and June 2023 in a real-world observational setting at University Hospital Heidelberg and Charité - Universitätsmedizin Berlin, and underwent a chest MRI before (MRI1, baseline MRI; mean time before therapy initiation 5.3 ± 4.8 months) and at least one subsequent MRI in the first (MRI2), second (MRI3) third year (MRI4) and/or fourth year (MRI5) on LUM/IVA, were included. On average, patients underwent 3.5 ± 1.1 MRI examinations (mean interval 12.0 ± 2.6 months, mean follow-up time 2.6 ± 1.1 years, maximum follow-up duration 4 years) (Table 1, Supplemental Figure E1, Supplemental Table E1-E3). Clinical data such as anthropometric data, spirometry, multiple-breath washout (MBW), SCC and throat swabs were obtained at MRI visits (mean time difference to MRI 5.6 ± 17.0 days) (Table 1). Spirometry (MasterScreen Body, E. Jaeger, Hoechberg, Germany) was performed in 32 children according to standards of the ERS/ATS to determine ppFEV1 [25, 26]. Nitrogen MBW (Exhalyzer^®^ D and Spiroware^®^ [analysis with version 3.2.1], Eco Medics AG, Duernten, Switzerland) was performed in 35 children by certified MBW operators to determine LCI [14, 27]. All children received symptomatic mucolytic therapy as per European standard and detailed in Table 1 [28]. Inhaled mucolytic therapy was initiated in one patient between MRI1 and MRI2 and in another patient between MRI1 and MRI3; the latter patient did not undergo MRI2. No further changes in concomitant inhaled therapy occurred during the observation period. Patients who changed therapy regime to ETI were excluded from this timepoint on (*n* = 34). Of note, some patients (*n* = 32) included in the present cohort were also included in our previous report on the effects of LUM/IVA on paranasal sinus abnormalities [29]. However, longitudinal changes in lung MRI abnormalities during long-term LUM/IVA therapy were not assessed in those studies, and none of the patients were included in the previously published randomized trial [10, 14, 16, 29].

**Table 1:** Patient characteristics at first magnetic resonance imaging (MRI1)

*n*=	49
Age (y)	5.0 ± 3.5
Sex (m/f)	26/23
Height (cm)	109.5 ± 24.1
Height z-score	-0.4 ± 1.3
Weight (kg)	19.8 ± 9.8
Weight z-score	-0.4 ± 1.0
*CFTR* genotype, *n* (%)	
F508del/F508del	49 (100)
Pancreatic insufficiency, *n* (%)	49 (100)
Therapy, *n* (%)	
Inhaled saline	49 (100)
- Isotonic	1 (2)
- Hypertonic	48 (98)
Inhaled mucolytics	22 (45)
Microbiology, *n* (%)	48 (98)
Intermittent P. aeruginosa	2 (4)
Chronic P. aeruginosa	0 (0)

Data presented as mean ± standard deviation, and absolute and relative numbers, respectively

*CFTR*  cystic fibrosis transmembrane conductance regulator, *P.* Pseudomonas

### Magnetic resonance imaging

Standardized MRI of the chest was performed using similar 1.5T scanners and identical protocol (Magnetom Avanto and Magnetom Aera, Siemens Healthcare, Erlangen, Germany) as previously described [13, 14, 20, 21, 30–32]. All subjects ≤ 5 years were routinely sedated with oral or rectal chloral hydrate (100 mg/kg body weight, maximum dose 2 g), and monitored by MRI-compatible pulse oximetry as previously described [12, 16]. All MRI were assessed in consensus by one reader with more than 15 years (MOW) and one reader with more than seven years (LW) of experience in MRI of patients with CF using the validated chest MRI scoring system. Both readers were blinded to clinical information, treatment duration, MRI examination dates, and chronological image order. Of note, in 5 children at MRI1, 4 children at MRI2, 4 children at MRI3, and 2 children at MRI4, perfusion imaging was not performed. The chest MRI score assesses main morphological and perfusion abnormalities, with scores of 0 for no abnormality, 1 referring to abnormalities affecting < 50% of the respective lung lobe, and 2 when abnormalities affected ≥ 50% of the respective lung lobe [8, 10–12, 14–21, 33]. In examinations without perfusion imaging, only the MRI morphology score was calculated; neither an MRI perfusion score nor an MRI global score was determined. For more information see the online data supplement.

### Statistical analyses

Data are presented as mean±standard deviation. For longitudinal intraindividual analysis ANOVA mixed-effects analysis with Greenhouse-Geisser correction and for group comparison (e.g. preschool age children vs. school age children) the Mann-Whitney test was used. Longitudinal paired prevalence data were analysed using the McNemar test. For categorical variables, Fisher’s exact test was used for comparisons between groups (e.g. preschool age children vs. school age children). The Spearman correlation coefficient r was calculated for chest MRI scores vs. clinical data, and interpreted as follows: 0.10–0.39 = weak, 0.40–0.69 = moderate, 0.70–0.89 = strong, 0.90-1.00 = very strong [34]. Tests were performed with Prism (version 10.4.2, GraphPad Software, San Diego, CA, USA), except for the McNemar test, which was calculated in Microsoft Excel (version 16.112.4, Redmond, Washington, USA). A p-value < 0.05 was considered statistically significant. Given the exploratory and hypothesis-generating nature of these analyses, p-values were not adjusted for multiple comparisons.

## Results

### Mid-term improvements of chest MRI scores under LUM/IVA treatment

**Fig. 1:**
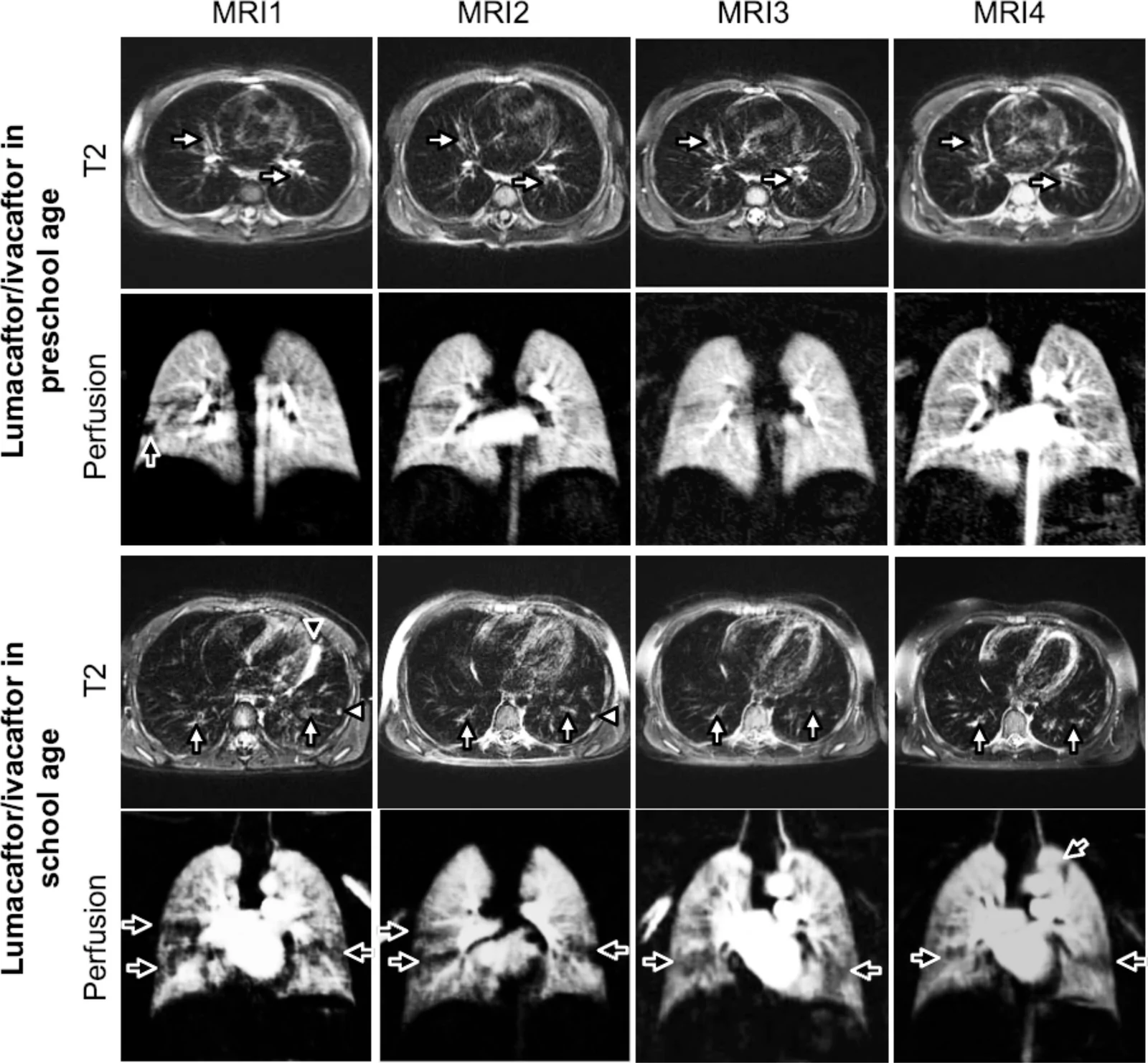
Representative examples of longitudinal magnetic resonance imaging of the chest before (MRI1) and under therapy with lumacaftor/ivacaftor (MRI2-4) in children with cystic fibrosis starting lumacaftor/ivacaftor therapy in preschool and school age. Bronchiectasis/wall thickening is indicated by white arrows, mucus plugging by white arrowheads, consolidations by black arrowheads and perfusion defects by black arrows. Of note, no MRI5 under lumacaftor/ivacaftor therapy was performed for the representative patients shown

**Fig. 2:**
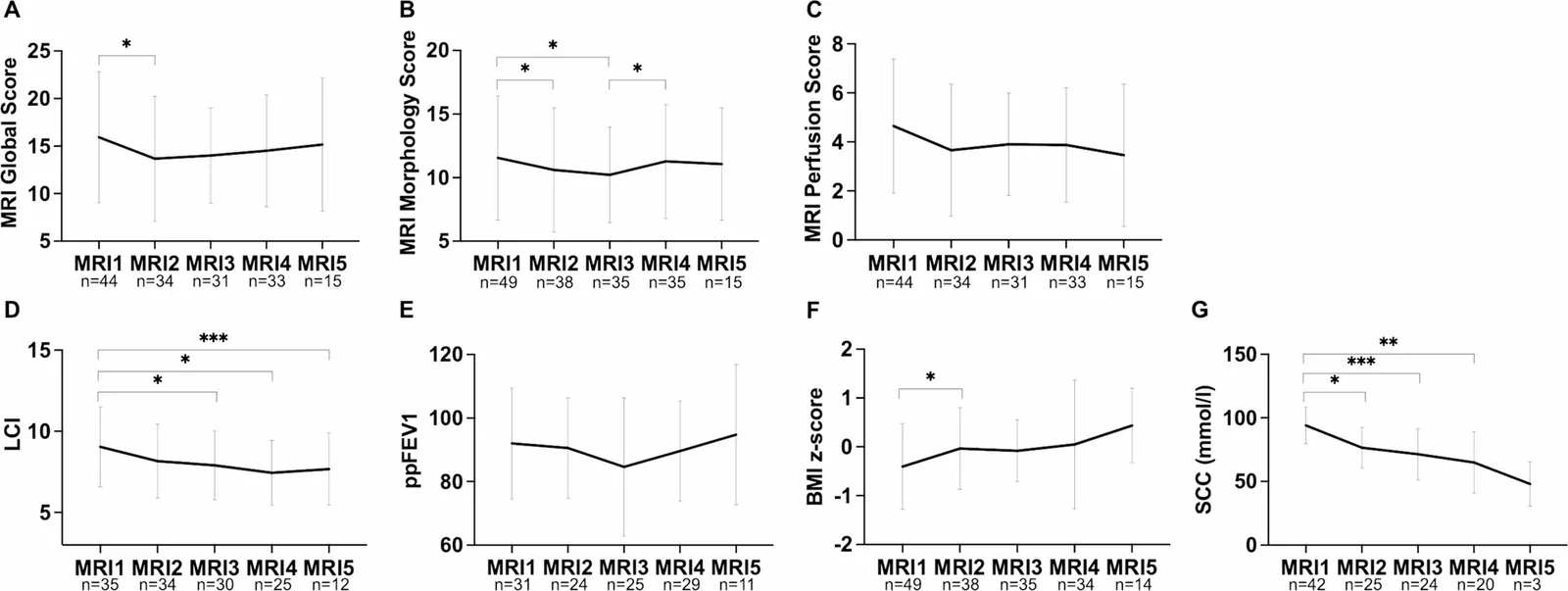
Chest magnetic resonance imaging (MRI) score, lung function test, body mass index z-score and sweat test before (MRI1) under therapy with lumacaftor/ivacaftor (MRI2-5) in children with cystic fibrosis. The chest MRI global score (**A**), chest MRI morphology score (**B**) and chest MRI perfusion scores (**C**), lung clearance index (LCI) (**D**), percent predicted forced expiratory volume in 1s (ppFEV1) (**E**), body mass index (BMI) z-score (**F**) and sweat chloride concentration (SCC) (**G**) were grouped by MRI timepoints. The mean is indicated by a line and whiskers mark the standard deviation. The number of examinations (n) is given for each MRI timepoint. *p < 0.05, **p < 0.01, ***p < 0.001. No statistical comparison was performed for sweat chloride concentration at MRI5 because data were available for only​ ​three patients (*n* = 3)

At MRI1, morphological lung abnormalities were present in all children with CF (100%) with bronchiectasis/wall thickening being the most prevalent (100%) and mucus plugging being the second most prevalent (90%) morphological lung abnormalities. Perfusion abnormalities were present in 86% of children with CF. The prevalence of lung abnormalities was stable from MRI1 to MRI2 (*p* = 0.180–0.999) (Fig. 1; Table 2). The mucus plugging subscore tended to decrease from MRI1 to MRI2 (3.4 ± 2.1 vs. 2.8 ± 2.2, *p* = 0.095), while the subscores for the other lung abnormalities remained stable (*p* = 0.247–0.999). MRI global and morphology score decreased from MRI1 to MRI2 (15.9 ± 6.8 vs. 13.7 ± 6.5, *p* < 0.05 and 11.6 ± 4.8 vs. 10.6 ± 4.8, *p* < 0.05; respectively) and MRI perfusion score tended to decrease (4.7 ± 2.7 vs. 3.7 ± 2.6, *p* = 0.200) (Figs. 1 and 2; Table 2, Supplemental Table E4). MRI global and morphology scores at MRI1 were lower in children starting therapy at preschool age than in children starting therapy at school age (14.3 ± 6.4 vs. 18.0 ± 6.7, *p* < 0.05; 10.0 ± 4.2 vs.13.0 ± 4.9, *p* < 0.05). In children starting therapy at preschool age, the MRI global score remained stable between MRI1 and MRI2 (14.3 ± 6.4 vs. 12.7 ± 6.0, *p* = 0.526), whereas it decreased in children starting therapy at school age (18.0 ± 6.7 vs.14.7 ± 6.7, *p* < 0.05) (Supplemental Table E5 and E6).

**Table 2:** Chest magnetic resonance imaging (MRI) scores in children with cystic fibrosis before (MRI1) and after start of lumacaftor/ivacaftor therapy (MRI2-5) in preschool and school age

	MRI1	MRI2	MRI3	MRI4	MRI5
Patients, *n*	49	38	35	35	15
Bronchiectasis/ Wall thickening					
Prevalence, *n* (%)	49 (100)	38 (100)	35 (100)	35 (100)	15 (100)
Subscore	6.1 ± 1.6	6.1 ± 1.3	6.1 ± 1.0	6.3 ± 1.1	6.4 ± 1.1
Mucus plugging					
Prevalence, *n* (%)	44 (90)	32 (84)	28 (80)	32 (91)	14 (93)
Subscore	3.4 ± 2.1	2.8 ± 2.2	2.9 ± 2.1	3.1 ± 2.0	3.0 ± 1.9
Abcesses/ Sacculations					
Prevalence, *n* (%)	5 (10)	5 (13)	2 (6)	3 (8)	1 (7)
Subscore	0.2 ± 0.9	0.2 ± 0.4	0.1 ± 0.2	0.1 ± 0.5	0.2 ± 0.7
Consolidation					
Prevalence, *n* (%)	17 (35)	11 (29)	5 (14)	10 (29)	4 (27)
Subscore	0.6 ± 0.9	0.4 ± 0.7	0.2 ± 0.6	0.4 ± 0.6	0.3 ± 0.6
Pleural findings					
Prevalence, *n* (%)	32 (65)	22 (58)	16 (46)*	20 (57)	9 (60)
Subscore	1.3 ± 1.4	1.1 ± 1.3	1.0 ± 1.4	1.4 ± 1.5	1.1 ± 1.1
MRI morphology score	11.6 ± 4.8	10.6 ± 4.8*	10.3 ± 3.9*	11.3 ± 4.5^**†**^	11.1 ± 4.3
MRI perfusion score					
Prevalence, *n* (%)	38 (86)	27 (79)	28 (90)	29 (87)	12 (80)
Score	4.7 ± 2.7	3.7 ± 2.6	4.1 ± 2.1	3.9 ± 2.3	3.5 ± 2.8
MRI global score	15.9 ± 6.8	13.7 ± 6.5*	14.2 ± 5.3	14.5 ± 5.8	15.2 ± 6.7

Prevalence presented as n (%) on a per-patient basis and scores as mean ± standard deviation. In examinations without perfusion imaging (MRI1: *n* = 5; MRI2: *n* = 4; MRI3: *n* = 4; MRI4: *n* = 2), only the MRI morphology score was calculated; no MRI perfusion or global score was determined

*p < 0.05 vs. MRI1, ^**†**^*p* < 0.05 vs. previous MRI

### Long-term stabilization of MRI perfusion score but progression of MRI morphology score during the third year of LUM/IVA treatment

Prevalence of consolidations tended to be lower in MRI3 compared to MRI1 (35% vs. 14%; *p* = 0.146, *n* = 35 pairs, b = 9, c = 3) and tended to increase again from MRI3 to MRI4 (14% vs. 29%; *p* = 0.125; *n* = 28 pairs, b = 0, c = 4). Prevalence of pleural findings was lower at MRI3 compared with MRI1 (65% vs. 46%; *p* < 0.05, *n* = 35 pairs, b = 8, c = 1) and remained stable thereafter (MRI3 vs. MRI4: *p* > 0.999, *n* = 28 pairs, b = 1, c = 2; MRI4 vs. MRI5: *p* > 0.999, *n* = 15 pairs, b = 0, c = 1) at the level of MRI1 (MRI4 vs. MRI1: *p* = 0.508, *n* = 35 pairs, b = 6, c = 3; MRI5 vs. MRI1: *p* = 0.688; *n* = 15 pairs, b = 4, c = 2). Prevalences of the other lung abnormalities remained stable from MRI2 through MRI5 (*p* = 0.219–0.999) and were at the level of MRI1 from MRI3 to MRI5 (*p* = 0.289–0.999 vs. MRI1). The abnormality subscores remained stable from MRI2 through MRI5 (*p* = 0.180–0.999) and were at the level of MRI1 from MRI3 to MRI5 (*p* = 0.326–0.992 vs. MRI1). MRI global score was relatively stable from MRI2 through MRI5 (MRI5: *n* = 15; *p* = 0.308–0.958). MRI morphology score was lower at MRI3 compared to MRI1 (10.3 ± 3.9 vs. 11.6 ± 4.8, *p* < 0.05), increased from MRI3 to MRI4 (10.3 ± 3.9 vs. 11.3 ± 4.5, *p* < 0.05), and was at the level of MRI1 at MRI4 and MRI5 (MRI5: *n* = 15; *p* = 0.990 and *p* = 0.999, respectively, vs. MRI1). The MRI perfusion score remained stable from MRI2 through MRI5 (MRI5: *n* = 15; *p* = 0.992–0.999) (Figs. 1 and 2; Table 2).

### Lung clearance index and sweat chloride concentration improve under LUM/IVA treatment

LCI was stable between MRI1 and MRI2 (9.0 ± 2.4 vs. 8.2 ± 2.2, *p* = 0.253) and from MRI3 onward lower compared to MRI1 (*p* < 0.05 vs. MRI1). ppFEV1 was stable from MRI1 through MRI5 (MRI5: *n* = 12; *p* = 0.355–0.999). BMI z-score increased from MRI1 to MRI2 (-0.4 ± 0.9 vs. 0.0 ± 0.8, *p* < 0.05), was stable from MRI2 through MRI5 (MRI5: *n* = 14; *p* = 0.903–0.993) and at the level of MRI1 at MRI3, MRI4 and MRI5 (MRI5: *n* = 14; *p* = 0.152–0.517 vs. MRI1). SCC decreased from MRI1 to MRI2 (94.1 ± 14.2 mmol/L vs. 76.5 ± 15.5, *p* < 0.05), and remained lower at MRI3 and MRI4 compared with MRI1 (*p* < 0.05 vs. MRI1). At MRI5, SCC was available in only three children and was therefore not interpreted (Fig. 2; Table 2).

### Changes in chest MRI scores in the first year under LUM/IVA treatment correlate with changes in lung clearance index

**Fig. 3:**
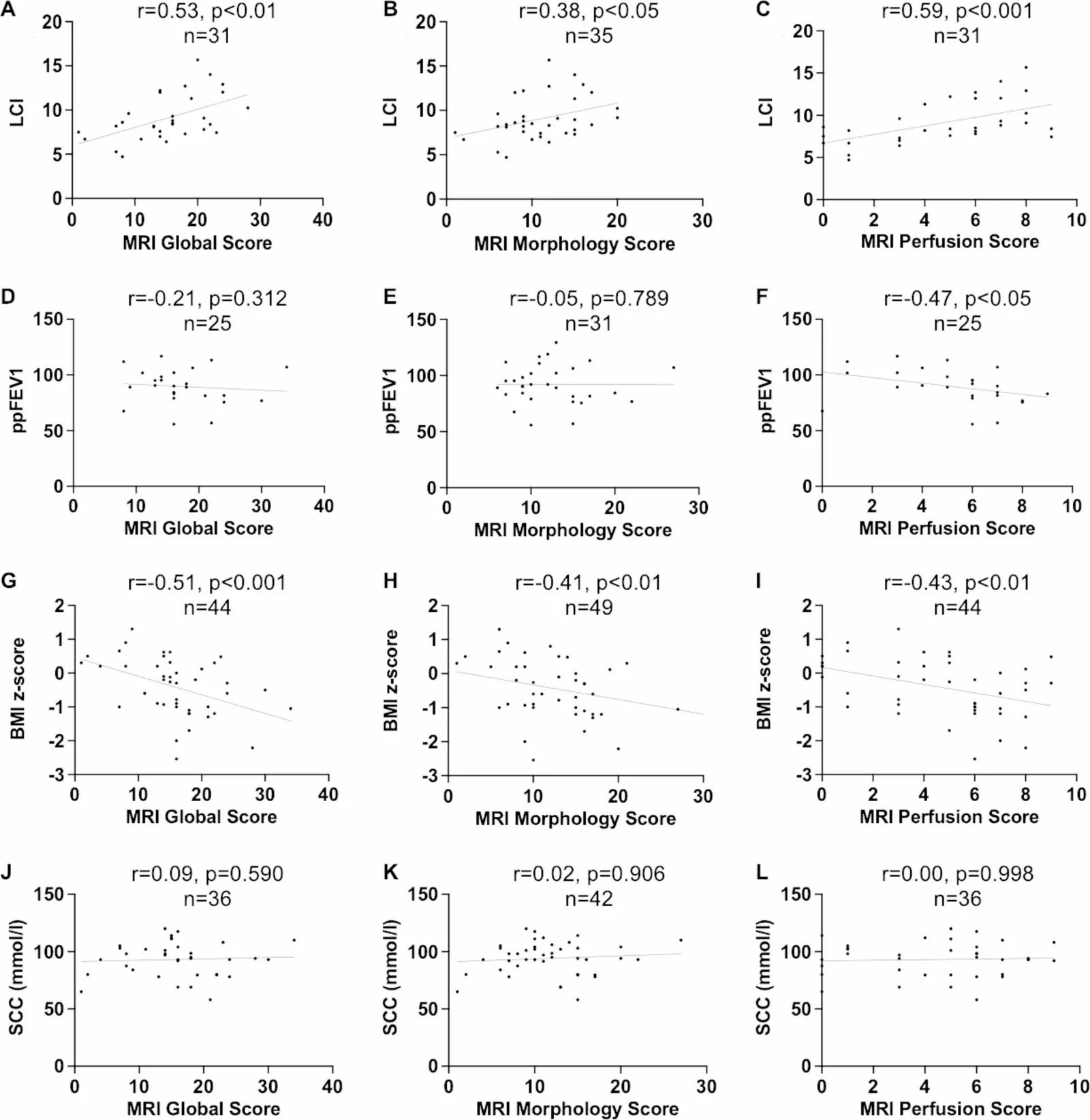
Correlation of chest magnetic resonance imaging (MRI) score with lung function test, body mass index (BMI) and sweat test at MRI1. Spearman correlation coefficient r, *P*-values and sample sizes (n) are shown for the chest MRI global score (**A**, **D**, **G**, **J**), chest MRI morphology score (**B**, **E**, **H**, K), and chest MRI perfusion score (**C**, **F**, **I**, **L**) vs. lung clearance index (**A**-**C**), percent predicted forced expiratory volume in 1s (FEV1) (**D**-**F**), BMI z-score (**G**-**I**) and sweat chloride concentration (SCC) (**J**-**L**) at MRI1

**Fig. 4:**
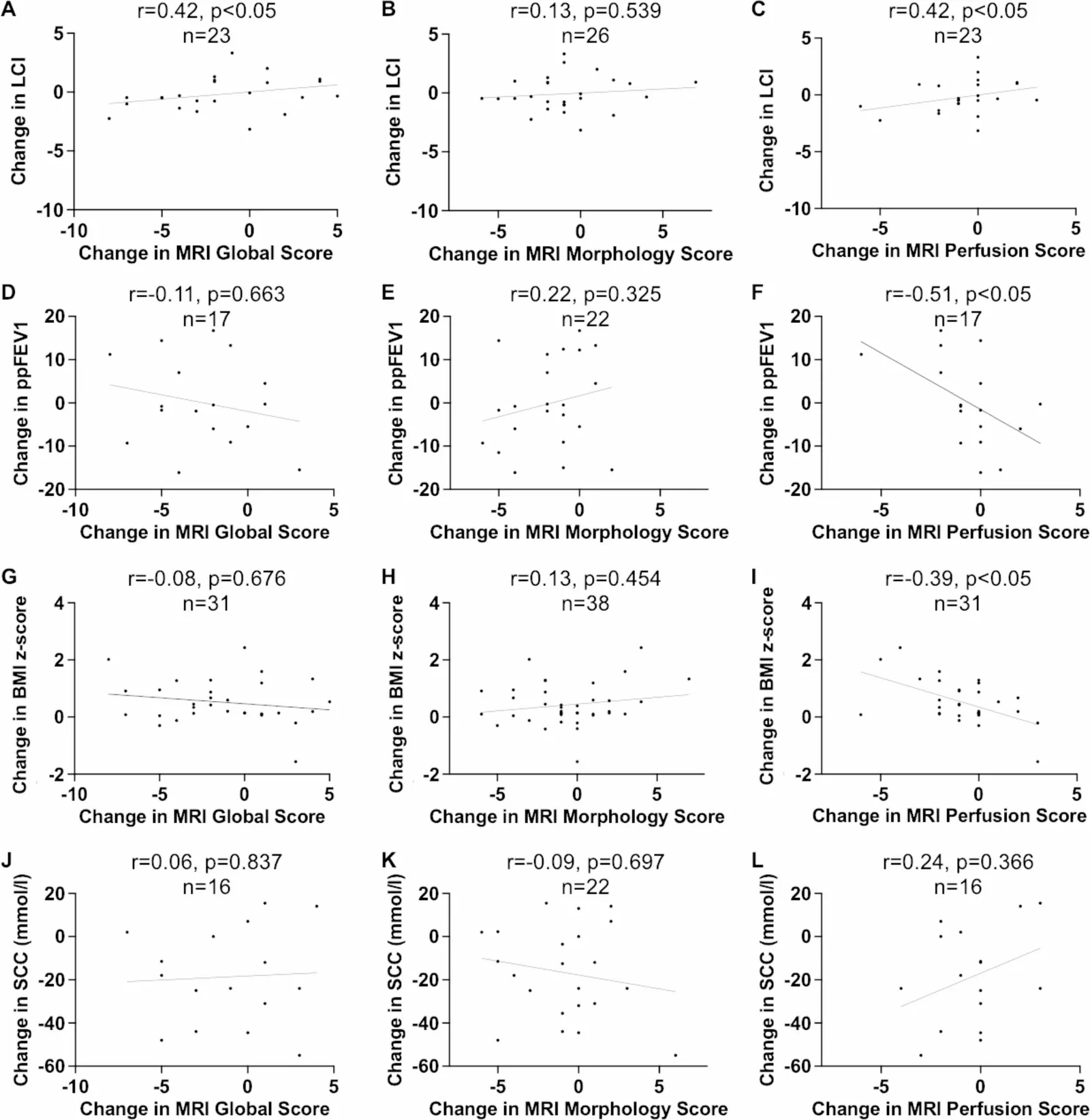
Correlation of changes in chest magnetic resonance imaging (MRI) score between MRI1 and MRI2 with changes in lung function test, body mass index (BMI) and sweat chloride concentration (SCC) between MRI1 and MRI2. Spearman correlation coefficient r, *P*-values and sample sizes (n) are shown for the chest MRI global score (**A**, **D**, **G**, **J**), chest MRI morphology score (**B**, **E**, **H**, **K**), and chest MRI perfusion score (**C**, **F**, **I**, **L**) vs. lung clearance index (**A**-**C**), percent predicted forced expiratory volume in 1s (FEV1) (**D**-**F**), BMI z-score (**G**-**I**) and sweat chloride concentration (SCC) (**J**-**L**) considering the difference between MRI1 and MRI2

At MRI1, chest MRI scores showed weak correlations with age (*r* = 0.28–0.35, *p* < 0.05), moderate inverse correlations with BMI z-score (*r*=-0.41 to -0.51, *p* < 0.05) and weak to moderate correlations with LCI (*r* = 0.38–0.59, *p* < 0.05) (Fig. 3, Supplemental Figure E2). MRI perfusion score correlated moderately negatively with ppFEV1 (*r*=-0.47, *p* < 0.05). SCC did not correlate with chest MRI scores (*r* = 0.00-0.09, *p* = 0.590–0.998) (Fig. 3). Improvements in MRI global and MRI perfusion score between MRI1 and MRI2 correlated weakly to moderately with improvements in LCI (both *r* = 0.42, *p* < 0.05), and improvements in MRI perfusion score between MRI1 and MRI2 with improvements in ppFEV1 and BMI z-score (*r*=-0.51, *p* < 0.05 and *r*=-0.39, *p* < 0.05, respectively) (Fig. 4). Changes in chest MRI perfusion score from MRI1 to the last available follow-up MRI correlated inversely with corresponding changes in ppFEV1 (*r*=-0.52, *p* < 0.05) (Supplemental Figure E3).

## Discussion

This is the first study assessing the long-term effects of treatment with LUM/IVA on prevalence and severity of lung abnormalities in preschool and school-age children with CF by consecutive annual chest MRI for up to four years. We show that in children with CF who are homozygous for the *F508del* mutation, lung abnormalities detected by chest MRI improve during the first year of LUM/IVA therapy, and afterwards stabilize in the second year of therapy. During the third year, however, morphological lung abnormalities as measured by the MRI morphology score increased again. In addition, we were able to demonstrate improvements in clinical parameters such as LCI and SCC after the start of LUM/IVA therapy.

At baseline, prevalence and severity of lung pathologies detected by MRI before LUM/IVA treatment are almost comparable to a previous MRI study in CFTR modulator-naïve children with CF, also reporting high prevalences of bronchiectasis/wall thickening (≥ 98%) and mucus plugging (≥ 70%), and mean chest MRI global scores between 11 and 14 score points in children with CF in preschool and school age, with higher MRI global scores in school-age than in preschool children. Also, clinical parameters such as BMI z-score, ppFEV1, LCI and SCC were in line with previous studies in patients with CF [8, 9, 20, 21, 35–37].

We demonstrate an improvement of morphological lung abnormalities and a trend towards improvement of functional lung abnormalities in preschool and school age children, as measured by the chest MRI scores, within the first year after initiation of LUM/IVA therapy. This was mainly driven by non-significant reductions of mucus plugging. The MRI global score decreased by 2.2 score points and the MRI morphology score by 1.0 score point during the first year of treatment, whereas the MRI perfusion score showed a numerical decrease of 1.0 score point that did not reach statistical significance. Importantly, previous reproducibility data in clinically stable adults with CF undergoing repeated chest MRI examinations at an interval of approximately one month demonstrated only minor mean changes between examinations (0.2 score points for the MRI global score, 0.3 for the MRI morphology score, and 0.0 for the MRI perfusion score), with limits of agreement of approximately ± 4 score points for the MRI global score and ± 2 score points for the MRI morphology and perfusion scores [15]. Thus, the mean changes observed in our cohort exceeded the average test-retest changes, although they remained within the corresponding limits of agreement, indicating modest treatment-associated improvements in MRI-detected lung abnormalities. Consistent with our findings, previous clinical trials also reported reductions of chest MRI scores in children with CF aged between two and five years treated for 48 weeks with LUM/IVA mainly due to reductions in consolidations, mucus plugging and perfusion abnormalities, and in patients with CF aged twelve years and older treated for 8–16 weeks with LUM/IVA [10, 22]. The mean numeric improvement in the MRI global score in our cohort of preschool and school age children (-2.2 score points) was slightly greater than the reduction of MRI global score in the above-mentioned study in preschool-aged children (two to five years) receiving LUM/IVA for 48 weeks (reduction of MRI global score: -1.7 score points) [22]. This difference may be explained by the broader age range of our cohort, additionally including school-age children with a higher baseline disease burden and consequently greater potential for measurable improvements in MRI scores. Consistent with this interpretation, an age-stratified analyses in our cohort showed that MRI global score remained stable during the first year in preschool children, whereas a reduction was observed in children starting therapy at school age. This finding raises the hypothesis that prevention of disease progression may be the predominant treatment effect in preschool children with mild baseline disease, whereas children with more advanced lung abnormalities may show greater measurable improvements in MRI scores. Nevertheless, the numerical reductions in MRI global score under LUM/IVA were considerably lower than the previously reported effects of ETI therapy during the first year of treatment in children six years and older and adults with CF (-3.5 to -11.4 score points), consistent with a higher effectiveness of this triple combination therapy [18, 33, 36].

During the second year of LUM/IVA therapy, chest MRI scores remained stable, reflecting stable morphological and functional lung abnormalities. The only exception was a lower prevalence of pleural findings compared with baseline; however, this isolated finding was not sustained at later follow-up time points and should therefore be considered exploratory. Thereafter, the MRI global score, representing the sum of the MRI morphology and perfusion scores, remained relatively stable through the fourth year of LUM/IVA therapy. However, the trajectories of the individual score components diverged. The MRI perfusion score remained stable, whereas the MRI morphology score increased during the third year of LUM/IVA therapy to the pre-treatment level, indicating a progression of morphological lung abnormalities. Given that modulator therapy reduces but does not fully resolve neutrophil-driven airway inflammation, the progression of morphological lung abnormalities during long-term therapy with LUM/IVA may reflect a persistent low-grade inflammatory response not fully controlled by treatment [38, 39]. A previous real-world computed tomography (CT) study in children with CF aged between six and eleven years similarly reported stability of the overall CT-based PRAGMA CF disease score over a two-year period under LUM/IVA but also demonstrated divergent changes in individual PRAGMA-CF components with a reduction in air trapping and an increase in bronchiectasis [35]. Together with our findings, this suggests that LUM/IVA may have differential effects on functional and morphological components of CF lung disease. To our knowledge, our study is the first to examine the effects of LUM/IVA over a treatment period of more than two years.

Moreover, we demonstrate that LUM/IVA therapy is associated with an initial improvement in BMI z-score within the first year of treatment, which was not sustained at later time points. In contrast, SCC improved within the first year and LCI within two years of treatment, with both remaining improved during longer-term follow-up. Improvements in BMI, LCI and SCC were also demonstrated in previous studies assessing the effects of LUM/IVA in children and adults with CF [8–10]. Also, the stability of ppFEV1 under LUM/IVA in our study is consistent with prior findings [40, 41]. This suggests that ppFEV1 may be a less sensitive marker compared to LCI in assessing mild treatment responses.

In addition, we showed that baseline chest MRI scores correlate particularly with age and LCI, which is consistent with previous studies [14]. This demonstrates that a higher disease severity on MRI occurs with older age as well as in patients with a worse lung function. Moreover, we could show that individual changes in MRI global and MRI perfusion score under LUM/IVA within the first year of therapy correlated with changes in LCI, supporting an association between imaging and functional lung parameters as previously published [14, 18]. However, the predominantly weak-to-moderate strength of these correlations suggests that MRI and LCI provide complementary rather than interchangeable information. While chest MRI provides regional information on morphological and perfusion abnormalities, LCI reflects global ventilation inhomogeneity. Thus, combining both measures may provide a more comprehensive characterization of lung disease than either parameter alone. Nevertheless, individual changes in MRI scores between the examinations did not correlate with either BMI or SCC.

Our study has some limitations. First, the number of patients included in our study was only moderate. However, it was difficult to maintain a large cohort on LUM/IVA over long-term follow-up because the more effective CFTR modulator combination ETI became available during the study period, resulting in treatment transition in a substantial proportion of patients. Importantly, transition to ETI was not delayed for the purpose of this observational study. Although baseline characteristics did not differ significantly between children with and without MRI5, the decreasing number of patients at later time points may still have introduced attrition-related selection bias. Therefore, particularly the findings at MRI5 should be interpreted with caution. In addition, analyses of changes from MRI1 to the last available follow-up MRI included variable follow-up intervals ranging from one to four years. Therefore, the changes between MRI1 to the last available follow-up MRI scores partly reflect differences in treatment duration and should also be interpreted with caution. Second, the study did not include an untreated control group. Therefore, the observed longitudinal changes cannot be unequivocally distinguished from the natural course of CF lung disease or attributed exclusively to LUM/IVA therapy. However, previous longitudinal MRI data provide a reference for the expected progression of CF lung abnormalities across childhood [21]. Third, certain clinical parameters, such as sweat testing, and perfusion imaging were not consistently available at all MRI time points, resulting in incomplete data for these parameters. No sensitivity analysis restricted to examinations with complete perfusion imaging was performed. Fourth, several MRI-related methodological limitations should be considered. MRI examinations were performed on two different clinical 1.5T scanners, which may have introduced scanner-related variability. However, each patient was examined on the same scanner at the same study site throughout longitudinal follow-up. In addition, MRI examinations were assessed by consensus, and cohort-specific inter-observer agreement was therefore not determined. Furthermore, sedation was routinely used in children ≤ 5 years of age, and sedation status could therefore change during longitudinal follow-up as children became older, potentially introducing additional variability between examinations. However, previous longitudinal studies did not find systematic differences at this transitional age [21]. Fifth, MRI1 was performed a mean of 5.3 ± 4.8 months before initiation of LUM/IVA therapy. Thus, minor changes in lung abnormalities during this pre-treatment interval cannot be excluded. Sixth, clinical assessments were performed a mean of 5.6 ± 17.0 days from the corresponding MRI examination. Thus, temporal differences between imaging and clinical assessments may have introduced additional variability into the MRI–clinical correlation analyses. Seventh, although no interruptions of LUM/IVA treatment were documented, quantitative adherence measures, such as pharmacy refill data or medication possession ratios, were not available. Thus, variations in adherence cannot be excluded as a potential contributor to the longitudinal changes, including the increase in morphological abnormalities during the third year of therapy. Eighth, although concomitant inhaled therapy remained largely unchanged during follow-up, inhaled mucolytic therapy was initiated in one patient between MRI1 and MRI2 and in another patient between MRI1 and MRI3. Therefore, a contribution of these treatment changes to the individual MRI changes observed in these patients cannot be excluded. Nineth, no adjustment for multiple comparisons was applied because the correlation analyses and secondary longitudinal comparisons were exploratory. Therefore, individual statistically significant associations should be interpreted with caution and require confirmation in independent cohorts.

In conclusion, our study demonstrates that LUM/IVA therapy in preschool- and school-age children with CF is associated with an initial improvement in lung abnormalities during the first year, followed by stabilization in the second year and a subsequent increase in morphological abnormalities toward pre-treatment levels during longer-term follow-up. LCI and SCC improved during the first or second year and remained improved during follow-up, whereas BMI z-score showed only an initial improvement during the first year, and ppFEV1 remained stable throughout. These findings suggest that LUM/IVA may be associated with delayed natural progression of CF lung disease during the first years of therapy. Moreover, our findings support MRI as a non-invasive and radiation-free imaging modality for longitudinal assessment of morphological and perfusion abnormalities under modulator therapy.

## Supplementary Information

Supplementary Material 1.

## Data Availability

The datasets generated and/or analyzed during the current study are not publicly available due to privacy and ethical restrictions but are available from the corresponding author on reasonable request and subject to approval by the local ethics committee and applicable data protection regulations.
