# Supplementary material for: Long-term effects of lumacaftor/ivacaftor on lung abnormalities detected by magnetic resonance imaging in children with cystic fibrosis
Source: Mol Cell Pediatr. 2026 Nov 10;13:55. doi: 10.1186/s40348-026-00268-7 (PMC13751989; doi:10.1186/s40348-026-00268-7)
Supplement: Supplementary file 1 [file 40348_2026_268_MOESM1_ESM.docx]

**Long-Term Effects of Lumacaftor/Ivacaftor on Lung Abnormalities Detected by Magnetic Resonance Imaging in Children with Cystic Fibrosis**

Lena Wucherpfennig, Monika Eichinger, Mirjam Stahl, Simon Y. Graeber, Shengkai Zhao, Jaehi Chung, Susanne Hämmerling, Jens-Peter Schenk, Abdulsattar Alrajab, Rory E. Morty, Julian Berges, Hans-Ulrich Kauczor, Marcus A. Mall, Mark O. Wielpütz, Eva Steinke^*^, Olaf Sommerburg^*^

** These authors contributed equally as senior authors.*

*Online Data Supplement*

**SUPPLEMENTAL METHODS**

**Magnetic resonance imaging**

MRI of the chest was performed using two clinical 1.5T scanners: Magnetom Avanto (Siemens Healthineers, Erlangen, Germany) at University Hospital Heidelberg (33 patients; 126 MRI examinations) and Magnetom Aera (Siemens Healthineers, Erlangen, Germany) at Charité – Universitätsmedizin Berlin (16 patients; 46 MRI examinations). Each patient was examined at the same study site and on the same scanner throughout longitudinal follow-up, with no scanner or site changes. All subjects ≤5 years were routinely sedated with oral or rectal chloral hydrate (100 mg/kg body weight, maximum dose of 2 g) and monitored by MRI compatible pulse oximetry as previously described [E1, E2]. For morphological chest imaging, T1 and T2-weighted sequences were used [E2-E7]. A T1-weighted turbo spin echo (TSE) sequence in free breathing was used before and after administration of intravenous contrast material. The T2- weighted sequence used was a “Half-Fourier single-shot turbo spin-echo” (HASTE) sequence with navigator-triggering in free breathing (PACE). Four-dimensional lung perfusion imaging was performed with intravenous injection of Gadolinium-based contrast material (0.1 mmol/kg body weight of Dotarem, Guerbet AG, Zurich, Switzerland or Gadovist, Bayer Schering AG, Germany) at a rate of 3-5 ml/s. Note that in infants contrast material was not routinely used due to applicable prescription restrictions.

All MRI were assessed in consensus by one reader with more than 15 years (MOW) and one reader with more than seven years (LW) of experience in MRI of patients with CF using the validated chest MRI scoring system. The chest MRI score consists of a morphology score assessing structural changes, a perfusion score assessing perfusion abnormalities, and a global score. The chest MRI morphology score is composed of subscores for the following structural changes: i) bronchial wall abnormalities, i.e. wall thickening and/or bronchiectasis; ii) mucus plugging; iii) sacculations and abscesses; iv) consolidations; and v) pleural reaction including effusion. The extent of abnormalities for each morphology subscore and for perfusion was rated in each lobe as 0 (no abnormality), 1 (<50% of the lobe involved), or 2 (≥50% of the lobe involved). The MRI global score results from the sum of the morphology and perfusion score. The maximal chest MRI score is 72 [E8].

**SUPPLEMENTAL RESULTS**

**Comparison of baseline characteristics in children with and without MRI5**

At MRI1, children who underwent MRI5 (n=15) and those without MRI5 (n=34) showed no statistically significant differences between groups in age, sex, height, weight, treatment, *Pseudomonas aeruginosa* infection status, or chest MRI scores (Supplemental Table E2).

**Comparison of baseline characteristics according to mode of diagnosis**

At MRI1, children diagnosed based on newborn screening (n=22) were younger, shorter, and lighter than children diagnosed based on clinical symptoms (n=27). However, height and weight z-scores were comparable between both groups, and no statistically significant differences were observed in sex, treatment, *Pseudomonas aeruginosa* infection status, or chest MRI scores (Supplemental Table E3).

**Changes in concomitant inhaled therapy during follow-up**

In the patient in whom inhaled mucolytic therapy was initiated between MRI1 and MRI2, the MRI global score remained unchanged at 28 score points. In the second patient, who did not undergo MRI2 and initiated inhaled mucolytic therapy between MRI1 and MRI3, the MRI global score decreased from 15 to 13 score points.

**SUPPLEMENTAL REFERENCES**

E1. Stahl M, Wielpütz MO, Ricklefs I, Dopfer C, Barth S, Schlegtendal A, et al. Preventive Inhalation of Hypertonic Saline in Infants with Cystic Fibrosis (PRESIS). A Randomized, Double-Blind, Controlled Study. Am J Respir Crit Care Med 2019; 199: 1238-1248.

E2. Wielpütz MO, Puderbach M, Kopp-Schneider A, Stahl M, Fritzsching E, Sommerburg O, et al. Magnetic resonance imaging detects changes in structure and perfusion, and response to therapy in early cystic fibrosis lung disease. Am J Respir Crit Care Med 2014; 189: 956-965.

E3. Stahl M, Wielpütz MO, Graeber SY, Joachim C, Sommerburg O, Kauczor HU, et al. Comparison of Lung Clearance Index and Magnetic Resonance Imaging for Assessment of Lung Disease in Children with Cystic Fibrosis. Am J Respir Crit Care Med 2017; 195: 349-359.

E4. Wielpütz MO, Eichinger M, Wege S, Eberhardt R, Mall MA, Kauczor HU, et al. Midterm Reproducibility of Chest Magnetic Resonance Imaging in Adults with Clinically Stable Cystic Fibrosis and Chronic Obstructive Pulmonary Disease. Am J Respir Crit Care Med 2019; 200: 103-107.

E5. Puderbach M, Eichinger M, Haeselbarth J, Ley S, Kopp-Schneider A, Tuengerthal S, et al. Assessment of morphological MRI for pulmonary changes in cystic fibrosis (CF) patients: comparison to thin-section CT and chest x-ray. Invest Radiol 2007; 42: 715-725.

E6. Puderbach M, Eichinger M, Gahr J, Ley S, Tuengerthal S, Schmahl A, et al. Proton MRI appearance of cystic fibrosis: comparison to CT. Eur Radiol 2007; 17: 716-724.

E7. Eichinger M, Heussel CP, Kauczor HU, Tiddens H, Puderbach M. Computed tomography and magnetic resonance imaging in cystic fibrosis lung disease. J Magn Reson Imaging 2010; 32: 1370-1378.

E8. Eichinger M, Optazaite DE, Kopp-Schneider A, Hintze C, Biederer J, Niemann A, et al. Morphologic and functional scoring of cystic fibrosis lung disease using MRI. Eur J Radiol 2012; 81: 1321-1329.

**SUPPLEMENTAL FIGURES**

**Supplemental Figure E1. Study flowchart.** ETI = elexacaftor/tezacaftor/ivacaftor, LUM/IVA = lumacaftor/ivacaftor, MRI = magnetic resonance imaging.

**Supplemental Figure E2. Correlation of chest magnetic resonance imaging (MRI) score with age at MRI1.** Spearman correlation coefficient r, *P*-values and sample sizes (n) are shown for the chest MRI global score, chest MRI morphology score, and chest MRI perfusion score vs. age at MRI1.

**Supplemental Figure E3. Correlation of changes in chest magnetic resonance imaging (MRI) score from MRI1 to the last follow-up MRI with changes in lung function test, body mass index (BMI) and sweat chloride concentration (SCC) from MRI1 to the last follow-up MRI.** Spearman correlation coefficient r, *P*-values and sample sizes (n) are shown for the chest MRI global score (A, D, G, J), chest MRI morphology score (B, E, H, K), and chest MRI perfusion score (C, F, I, L) vs. lung clearance index (A-C), percent predicted forced expiratory volume in 1s (FEV1) (D-F), BMI z-score (G-I) and sweat chloride concentration (SCC) (J-L) considering differences from MRI1 to the last follow-up MRI.

**SUPPLEMENTAL TABLES**

**Supplemental Table E1. Patient characteristics at first magnetic resonance imaging (MRI1) grouped by age at therapy start.**

|  | **Lumacaftor/ivacaftor**  **from preschool age** | **Lumacaftor/ivacaftor**  **from school age** |
| --- | --- | --- |
| **n=** | 24 | 25 |
| **Age (y)** | 1.9±1.3 | 8.0±2.1*** |
| **Sex (m/f)** | 12/12 | 14/11 |
| **Height (cm)** | 89.0±13.6 | 129.1±12.9*** |
| *Height z-score* | -0.4±1.3 | -0.4±1.3 |
| **Weight (kg)** | 12.4±3.5 | 26.8±8.5*** |
| *Weight z-score* | -0.4±0.8 | -0.4±1.1 |
| ***CFTR* genotype, n (%)** |  |  |
| *F508del/F508del* | 24 (100) | 25 (100) |
| **Pancreatic insufficiency, n (%)** | 24 (100) | 25 (100) |
| **Therapy, n (%)** |  |  |
| *Inhaled lung saline* | 24 (100) | 25 (100) |
| - *Isotonic* | 1 (4) | 0 (0) |
| - *Hypertonic* | 23 (96) | 25 (100) |
| *Inhaled mucolytics* | 8 (33) | 14 (56) |
| **Microbiology, n (%)** | 23 (96) | 25 (100) |
| *Intermittent P. aeruginosa* | 0 (0) | 2 (8) |
| *Chronic P. aeruginosa* | 0 (0) | 0 (0) |

Data presented as mean ± standard deviation, and absolute and relative numbers, respectively. BMI = body mass index, *CFTR =* cystic fibrosis transmembrane conductance regulator, *P*. = *Pseudomonas*. ***p<0.001 vs. Lumacaftor/ivacaftor from preschool age.

**Supplemental Table E2. Patient characteristics at first magnetic resonance imaging (MRI1) in children with and without fifth MRI (MRI5).**

|  | **Children with MRI5** | **Children without MRI5** |
| --- | --- | --- |
| **n=** | 15 | 34 |
| **Age (y)** | 6.3±3.8 | 4.5±3.3 |
| **Sex (m/f)** | 10/5 | 16/18 |
| **Height (cm)** | 120.4±22.1 | 104.3±22.7 |
| *Height z-score* | -0.2±1.2 | -0.5±1.3 |
| **Weight (kg)** | 24.3±12.0 | 17.5±7.5 |
| *Weight z-score* | -0.3±1.0 | -0.5±1.0 |
| ***CFTR* genotype, n (%)** |  |  |
| *F508del/F508del* | 15 (100) | 34 (100) |
| **Pancreatic insufficiency, n (%)** | 15 (100) | 34 (100) |
| **Therapy, n (%)** |  |  |
| *Inhaled lung saline* | 15 (100) | 34 (100) |
| - *Isotonic* | 0 (0) | 1 (3) |
| - *Hypertonic* | 15 (100) | 33 (97) |
| *Inhaled mucolytics* | 8 (53) | 14 (41) |
| **Microbiology, n (%)** | 14 (93) | 34 (100) |
| *Intermittent P. aeruginosa* | 1 (7) | 1 (3) |
| *Chronic P. aeruginosa* | 0 (0) | 0 (0) |
| **MRI morphology score** | 11.5±3.3 | 11.6±5.4 |
| **MRI perfusion score** | 4.4±2.7 | 4.8±2.7 |
| **MRI global score** | 15.9±4.8 | 16.0±7.6 |

Data presented as mean ± standard deviation, and absolute and relative numbers, respectively. BMI = body mass index, *CFTR =* cystic fibrosis transmembrane conductance regulator, *P.* = *Pseudomonas*.

**Supplemental Table E3. Patient characteristics at first magnetic resonance imaging (MRI1), stratified by diagnosis based on newborn screening or clinical symptoms.**

|  | **Children diagnosed based on newborn screening** | **Children diagnosed based on clinical symptoms** |
| --- | --- | --- |
| **n=** | 22 | 27 |
| **Age (y)** | 3.3±3.0 | 6.7±3.2*** |
| **Sex (m/f)** | 9/13 | 17/10 |
| **Height (cm)** | 96.8±22.4 | 121.7±19.6** |
| *Height z-score* | -0.3±1.0 | -0.4±1.5 |
| **Weight (kg)** | 15.0±6.7 | 24.5±10.0** |
| *Weight z-score* | -0.4±0.7 | -0.3±1.1 |
| ***CFTR* genotype, n (%)** |  |  |
| *F508del/F508del* | 22 (100) | 27 (100) |
| **Pancreatic insufficiency, n (%)** | 22 (100) | 27 (100) |
| **Therapy, n (%)** |  |  |
| *Inhaled lung saline* | 22 (100) | 27 (100) |
| - *Isotonic* | 1 (5) | 0 (0) |
| - *Hypertonic* | 21 (95) | 27 (100) |
| *Inhaled mucolytics* | 7 (32) | 15 (56) |
| **Microbiology, n (%)** | 22 (100) | 26 (96) |
| *Intermittent P. aeruginosa* | 0 (0) | 2 (7) |
| *Chronic P. aeruginosa* | 0 (0) | 0 (0) |
| **MRI morphology score** | 10.5±4.2 | 12.4±5.2 |
| **MRI perfusion score** | 3.8±2.6 | 5.5±3.1 |
| **MRI global score** | 14.3±6.0 | 17.5±7.1 |

Data presented as mean ± standard deviation, and absolute and relative numbers, respectively. BMI = body mass index, *CFTR =* cystic fibrosis transmembrane conductance regulator, *P.* = *Pseudomonas*. **p<0.01 vs. children diagnosed based on newborn screening, ***p<0.001 vs. children diagnosed based on newborn screening.

**Supplemental Table E4. No statistical comparison was performed for sweat chloride concentration at MRI5 because datawere available for only three patients (n=3).**

|  | **MRI1** | **MRI2** | **MRI3** | **MRI4** | **MRI5** |
| --- | --- | --- | --- | --- | --- |
| **Multiple breath washout,** n (%) | 35 (71) | 34 (89) | 30 (86) | 25 (71) | 12 (80) |
| *LCI* | 9.0±2.4 | 8.2±2.2 | 7.9±2.1* | 7.4±2.0* | 7.7±2.1*** |
| **Spirometry,** n (%) | 31 (63) | 24 (63) | 25 (71) | 29 (83) | 11 (73) |
| *ppFEV1* | 93.2±16.2 | 92.6±14.4 | 85.0±21.6 | 89.6±15.5 | 94.8±21.0 |
| **Anthropometric data,** n | 49 (100) | 38 (100) | 35 (100) | 34 (97) | 14 (93) |
| *BMI z-score* | -0.4±1.0 | 0.0±0.8* | -0.1±0.6 | 0.0±1.3 | 0.4±0.7 |
| **Sweat chloride concentration,** n (%) | 42 (86) | 25 (66) | 24 (69) | 20 (57) | 3 (20) |
| *Sweat chloride concentration (mmol/l)* | 94.1±14.2 | 76.5±15.5* | 71.4±19.7*** | 65.0±23.5** | 48.0±14.2 |

Prevalence presented as n (%) on a per-patient basis and scores as mean ± standard deviation. *p<0.05 vs. MRI1, **p<0.01 vs. MRI1, ***p<0.001 vs. MRI1. No statistical comparison was performed for sweat chloride concentration at MRI5 because data were available for only three patients (n=3). BMI= body mass index, LCI = lung clearance index, ppFEV1 = percent predicted forced expiratory volume in 1s.

**Supplemental Table E5. Chest magnetic resonance imaging (MRI) scores and clinical data in children with cystic fibrosis before (MRI1) and after start of lumacaftor/ivacaftor therapy (MRI2-5) in preschool age.**

|  | **MRI1** | **MRI2** | **MRI3** | **MRI4** | **MRI5** |
| --- | --- | --- | --- | --- | --- |
| **Patients, n** | 24 | 19 | 15 | 15 | 7 |
| **Bronchiectasis/**  **Wall thickening** |  |  |  |  |  |
| Prevalence, n (%) | 24 (100) | 19 (100) | 15 (100) | 15 (100) | 7 (100) |
| Subscore | 5.5±1.7 | 5.8±1.5 | 5.7±0.8 | 5.9±0.8 | 6.0±0.3 |
| **Mucus plugging** |  |  |  |  |  |
| Prevalence, n (%) | 21 (88) | 15 (79) | 12 (80) | 13 (87) | 6 (86) |
| Subscore | 3.0±1.8 | 2.7±2.1 | 2.7±2.1 | 2.7±1.7 | 2.9±1.8 |
| **Abcesses/ Sacculations** |  |  |  |  |  |
| Prevalence, n (%) | 0 (0) | 0 (0) | 0 (0) | 0 (0) | 0 (0) |
| Subscore | 0.0±0.0 | 0.0±0.0 | 0.0±0.0 | 0.0±0.0 | 0.0±0.0 |
| **Consolidation** |  |  |  |  |  |
| Prevalence, n (%) | 7 (29) | 6 (32) | 1 (7) | 5 (33) | 0 (0) |
| Subscore | 0.5±0.8 | 0.5±0.8 | 0.1±0.2 | 0.3±0.5 | 0.0±0.0 |
| **Pleural findings** |  |  |  |  |  |
| Prevalence, n (%) | 15 (63) | 10 (53) | 5 (33) | 8 (53) | 4 (57) |
| Subscore | 1.1±1.2 | 0.9±1.0 | 0.6±1.0 | 1.0±1.2 | 0.7±0.7 |
| **MRI morphology score** | 10.0±4.2 | 9.9±4.4 | 9.1±2.8 | 10.0±2.9 | 9.6±2.3 |
| **MRI perfusion score** |  |  |  |  |  |
| Prevalence, n (%) | 19 (86) | 14 (82) | 13 (93) | 12 (86) | 5 (71) |
| Score | 4.3±3.0 | 3.1±2.5 | 3.3±1.9 | 3.3±2.0 | 2.2±2.0 |
| **MRI global score** | 14.3±6.4 | 12.7±6.0 | 12.6±3.9 | 12.9±3.9 | 12.3±3.5 |
| **Multiple breath washout,** n (%) | 16 (67) | 15 (79) | 12 (80) | 10 (67) | 4 (57) |
| *LCI* | 8.9±2.6 | 8.3±2.3 | 7.2±1.2 | 6.5±0.6 | 6.8±0.7 |
| **Spirometry,** n (%) | 6 (25) | 5 (26) | 6 (40) | 11 (73) | 3 (43) |
| *ppFEV1* | 86.7±9.6 | 89.7±7.8 | 90.4±6.8 | 91.3±15.0 | 98.6±11.7 |
| **Anthropometric data,** n | 24 (100) | 19 (100) | 15 (100) | 14 (93) | 7 (100) |
| *BMI z-score* | -0.4±0.9 | 0.0±0.9 | 0.0±0.5 | -0.1±0.6 | 0.5±0.2 |
| **Sweat chloride concentration,** n (%) | 20 (83) | 13 (68) | 11 (73) | 6 (40) | 1 (14) |
| *Sweat chloride concentration (mmol/l)* | 97.2±13.7 | 78.8±13.2 | 73.8±12.2** | 70.5±14.3* | 68.0±0.0 |

Prevalence presented as n (%) on a per-patient basis and scores as mean ± standard deviation. *p<0.05 vs. MRI1, **p<0.01 vs. MRI1. No statistical comparison was performed for sweat chloride concentration at MRI5 because data were available for only one patient (n=1). BMI= body mass index, LCI = lung clearance index, ppFEV1 = percent predicted forced expiratory volume in 1s.

**Supplemental Table E6. No statistical comparison was performed for sweat chlorideconcentration at MRI5 because data were available for only two patients (n=2).**

|  | **MRI1** | **MRI2** | **MRI3** | **MRI4** | **MRI5** |
| --- | --- | --- | --- | --- | --- |
| **Patients, n** | 25 | 19 | 20 | 20 | 8 |
| **Bronchiectasis/**  **Wall thickening** |  |  |  |  |  |
| Prevalence, n (%) | 25 (100) | 19 (100) | 20 (100) | 20 (100) | 8 (100) |
| Subscore | 6.7±1.3 | 6.4±1.1 | 6.4±1.1 | 6.6±1.4 | 6.8±1.4 |
| **Mucus plugging** |  |  |  |  |  |
| Prevalence, n (%) | 23 (92) | 17 (89) | 16 (80) | 19 (95) | 8 (100) |
| Subscore | 3.8±2.2 | 2.9±2.3 | 3.0±2.1 | 3.4±2.1 | 3.1±2.0^††^ |
| **Abcesses/ Sacculations** |  |  |  |  |  |
| Prevalence, n (%) | 5 (20) | 5 (26) | 2 (10) | 3 (15) | 1 (13) |
| Subscore | 0.4±1.2 | 0.3±0.6 | 0.1±0.3 | 0.3±0.6 | 0.4±1.0 |
| **Consolidation** |  |  |  |  |  |
| Prevalence, n (%) | 10 (40) | 5 (26) | 4 (20) | 5 (25) | 4 (50) |
| Subscore | 0.6±1.0 | 0.3±0.6 | 0.3±0.7 | 0.4±0.7 | 0.6±0.7 |
| **Pleural findings** |  |  |  |  |  |
| Prevalence, n (%) | 17 (68) | 12 (63) | 11 (55) | 12 (60) | 5 (63) |
| Subscore | 1.4±1.5 | 1.3±1.5 | 1.4±1.5 | 1.7±1.7 | 1.5±1.3 |
| **MRI morphology score** | 13.0±4.9 | 11.3±5.1 | 11.2±4.3 | 12.3±5.1 | 12.4±5.1 |
| **MRI perfusion score** |  |  |  |  |  |
| Prevalence, n (%) | 19 (86) | 13 (76) | 15 (88) | 17 (89) | 7 (88) |
| Score | 5.2±2.1 | 4.3±2.7* | 4.8±1.9 | 4.1±2.6 | 4.6±2.9 |
| **MRI global score** | 18.0±6.7 | 14.7±6.7* | 15.6±5.9 | 15.7±6.6 | 17.6±7.8 |
| **Multiple breath washout,** n (%) | 19 (76) | 19 (100) | 18 (90) | 15 (75) | 8 (100) |
| *LCI* | 9.2±2.3 | 8.1±2.2 | 8.4±2.4 | 8.0±2.3* | 8.0±2.4^***^ |
| **Spirometry,** n (%) | 25 (100) | 19 (100) | 19 (95) | 18 (90) | 8 (100) |
| *ppFEV1* | 93.3±18.3 | 90.8±16.9 | 82.8±23.8 | 88.6±15.8 | 89.6±21.4 |
| **Anthropometric data,** n | 25 (100) | 19 (100) | 20 (100) | 20 (100) | 7 (88) |
| *BMI z-score* | -0.4±0.8 | 0.0±0.7* | -0.1±0.7 | 0.2±1.6 | 0.4±0.8 |
| **Sweat chloride concentration,** n (%) | 22 (88) | 12 (63) | 13 (65) | 14 (70) | 2 (25) |
| *Sweat chloride concentration (mmol/l)* | 91.4±14.0 | 74.0±17.4* | 69.3±24.2*** | 62.6±26.1** | 60.0±8.0 |

Prevalence presented as n (%) on a per-patient basis and scores as mean ± standard deviation. *p<0.05 vs. MRI1, **p<0.01 vs. MRI1, ***p<0.001 vs. MRI1, ^††^p<0.01 vs. previous MRI. No statistical comparison was performed for sweat chloride concentration at MRI5 because data were available for only two patients (n=2). BMI= body mass index, LCI = lung clearance index, ppFEV1 = percent predicted forced expiratory volume in 1s.
